# Coronary Artery Disease in Patients Undergoing Hemodialysis: A Problem that Sounds the Alarm

**DOI:** 10.31083/j.rcm2506200

**Published:** 2024-05-30

**Authors:** Simona Barbuto, Lilio Hu, Chiara Abenavoli, Matilde Picotti, Gaetano La Manna, Luca De Nicola, Simonetta Genovesi, Michele Provenzano

**Affiliations:** ^1^Nephrology, Dialysis and Renal Transplant Unit, IRCCS Azienda Ospedaliero-Universitaria di Bologna, 40126 Bologna, Italy; ^2^Nephrology Unit, Department of Medical and Surgical Science (DIMEC), Alma Mater Studiorum University of Bologna, 40126 Bologna, Italy; ^3^Division of Nephrology, University of Campania “Luigi Vanvitelli”, 80137 Naples, Italy; ^4^School of Medicine and Surgery, Nephrology Clinic, University of Milano Bicocca, 20900 Monza, Italy; ^5^Istituto Auxologico Italiano, IRCCS, 20095 Milan, Italy

**Keywords:** chronic kidney disease, kidney failure, coronary artery disease, hemodialysis

## Abstract

Chronic kidney disease (CKD) is affecting more and more individuals over time. The importance of the increased prevalence is enhanced by the close association with the increased risk of poor individual outcomes such as death, fatal and non-fatal cardiovascular (CV) events and progression to end stage kidney disease (ESKD). ESKD requires replacement treatment such as hemodialysis (HD), a particular and complex context that unfortunately has been rarely considered in observational studies in the last few decades. The current perspective of HD as a bridge to kidney transplant requires greater attention from observational and experimental research both in the prevention and treatment of CV events in ESKD patients. We present a narrative review by performing a literature review to extrapolate the most significant articles exploring the CV risk, in particular coronary artery disease (CAD), in ESKD and evaluating possible innovative diagnostic and therapeutic tools in these patients. The risk of CAD increases linearly when the estimated glomerular filtration rate (eGFR) declines and reached the most significant level in ESKD patients. Several diagnostic techniques have been evaluated to predict CAD in ESKD such as laboratory tests (Troponin-T, N-terminal pro b-type natriuretic peptide, alkaline phosphatase), echocardiography and imaging techniques for vascular calcifications evaluation. Similarly, treatment is based on lifestyle changes, medical therapy and invasive techniques such as coronary artery bypass grafting (CABG) and percutaneous coronary intervention (PCI). Unfortunately in the literature there are no clear indications of the usefulness and validity of biomarkers and possible treatments in ESKD patients. Considering the ESKD weight in terms of prevalence and costs it is necessary to implement clinical research in order to develop prognostic reliable biomarkers for CV and CAD risk prediction, in patients with ESKD. It should be highlighted that HD is a peculiar setting that offers the opportunity to implement research and facilitates patient monitoring by favoring the design of clinical trials.

## 1. Introduction

The current guidelines define chronic kidney disease (CKD) as the presence of kidney structure abnormalities associated with an estimated glomerular filtration rate (eGFR) <60 mL/min and/or albuminuria or urinary sediment anomalies for at least 3 months [1]. From an epidemiological perspective, CKD is increasing in prevalence, affecting about 15% of the adult population in the United States (U.S.) and 13.4% of individuals worldwide [2, 3]. The importance of such evidence is due to the link between the presence of CKD and an increased risk of poor individual outcomes such as death, fatal and non-fatal cardiovascular (CV) events and progression to end stage kidney disease (ESKD), which often needs kidney replacement therapies like dialysis (hemodialysis, HD, or peritoneal dialysis, PD) or kidney transplantation. Hemodialysis was discovered and implemented between 1943 and 1945 by two brilliant scientists, Kolff and Scribner, and after a series of subsequentimprovements, it was circulated to thousands of patients worldwide, particularly in high income countries at the beginning [4]. This therapeutic strategy from one side was considered a miracle, which became routine, giving chance to patients with ESKD to survive for longer, compared to untreated patients. On the other hand, patients undergoing HD experience a low quality of life and high morbidity and mortality [5]. HD patients indeed suffer, often from fatigue, pain, cramps, feeling washed out after treatment, depression, and other social problems (related to inability to work or do free-time activities) [6]. Moreover, the more fearsome event that occurs in these patients is represented by fatal and non-fatal CV events. Mortality risk from CV causes in Kidney Failure (KF) patients is up to 500 times higher than patients without kidney disease who are the same age, being the principal specific CV causes related to coronary artery disease (CAD) and heart failure (HF) [7]. These impressive data should be considered together with the epidemiologic evidence that HD is the preferred kidney replacement therapy offered to patients worldwide and its need is continuously rising. It has been reported that a reduction in overall mortality risk over the past two decades in HD patients may be related to better individual patient care and management of risk factors [8]. This is a first positive step, butneeds future and further efforts from the scientific and medical community. This is particularly true if considering the very complex clinical context of HD characterized by high-risk patients and the high cost of the technique. What has been prompted in the recent past, is to change the approach to HD patients, namely to consider these patients not on an untreatable phase of their clinical history but as patients that need the best possible care of all risk factors and comorbidities [9]. A perspective that is gaining momentum due to the increasing number of patients undergoing HD and the improvement in kidney transplant which, on average is performed 2.5 years after dialysis initiation, thus leaving HD as the main bridge to kidney transplant rather than the last phase of patient’s life [10]. To improve CV prognosis in HD patients, an effort is needed in the context of observational and experimental research. It is well documented that CV risk scores do not properly work in HD patients who seem to have a CV risk predicted from different variables as compared to CKD patients [11]. In an experimental context, HD patients have long been excluded from randomized studies meaningwe do not have robust evidence about the more appropriate care [12]. To this aim, we present a narrative review reporting an overview of CV risk, particularly focused on CAD, in HD patients. The narration will encompass the role of prognostic risk factors of CV and CAD, the evidence of treatments that reduce this risk and potential future strategies to improve the management of CV risk in this high-risk setting.

## 2. CKD and CAD: Epidemiology and Pathophysiology

**Fig. 1.**
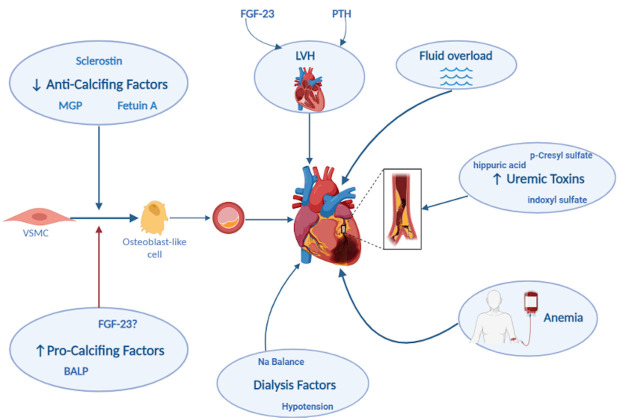
**Mechanisms of coronary artery disease (CAD) in patients with end stage kidney disease (ESKD).** MGP, Matrix-Gla protein; VSMC, vascular smooth muscle cell; FGF-23, fibroblast growth factor-23; BALP, bone alkaline phosphatase; Na, sodium; PTH, parathormone; LVH, left ventricular hypertrophy.

CKD is associated with an increased risk for CV disease and CAD [13]. As eGFR goes below 60 mL/min, the risk of CAD linearly increases with a relative risk almost doubled for eGFR of 45–60 mL/min and triplicated in those with eGFR of 15–44 mL/min as compared with patients without CKD [14]. Importantly, such a raised risk is independent from other traditional (such as hypertension, diabetes or dyslipidemia) and non-traditional (mineral bone disorder, CKD-related anemia, oxidative stress, inflammation, left ventricular hypertrophy) risk factors [15]. In ESKD, CAD risk is even higher due to the persistent presence of the same risk factors as earlier CKD stages and in addition to peculiar dialysis risk factors. The latter includes type and frequency of dialysis, intradialytic hypotension, myocardial stunning and continuous changes in extracellular and intravascular body volume [16] (Fig. 1). The overall prevalence of CAD in ESKD ranges from 15% to 37%, with a slightly higher percentage in HD as compared with PD patients and with increasing prevalence with age [17, 18, 19] (Table 1, Ref. [17, 18, 19]). In HD patients, cardiac disease accounts for 40% of death of which 8% are CAD-related [20]. There also seems to be an influence of ethnicity on the incidence of CAD in HD patients with a higher rate in Caucasian than Afro-American patients [21]. Frequently, in CKD and HD patients, coronary syndrome has an atypical presentation with only 41% experiencing chest pain, compared to 62% without CKD, and only 18% having an ST elevation at the electrocardiogram (ECG) evaluation compared to 33% without CKD. This unfortunately results in delayed diagnosis (only 20% diagnosed on admission) and a likely less appropriate treatment [22]. This clinical presentation, influenced by the presence of CKD, prompts a better understanding of how to monitor ESKD patients for CAD risk and how to improve risk prediction in a per se high-risk setting.

**Table 1.** **Observational studies regarding the prevalence of CAD in ESKD patients**.

Study	Population	Results
Soubassi, 2007 [17]	171 hemodialysis patients were examined with coronary angiography or combined dipyridamole-exercise thallium imaging	50 patients (29.2%) were clinically diagnosed with CAD. Using linear regression analysis, CAD was associated with the inadequacy of HD (r = –0.05, *p* < 0.0001), time on HD (r = 0.04, *p* = 0.012) and increasing age (r = 0.24, *p* < 0.001). The incidence of CAD in dialysis patients is significantly increased with age, male sex, obesity, time on dialysis, the presence of anemia, hyperhomocysteinemia and inadequacy of HD.
Soubassi, 2006 [18]	128 hemodialysis patients over 65 years old	48 patients (37%) were diagnosed with CAD by coronary angiography (n = 22) and dipyridamole stress test (n = 26). CAD incidence increases with age, male sex, diabetes, secondary hyperparathyroidism, hypertension, increased CRP, hyperhomocysteinemia, smoking, time on HD and inadequacy of HD.
Yao-Min Hung, 2018 [19]	1624 new onset ESRD patients	Patients undergoing HD had significantly higher risks of incidence of CAD, in comparison with patients undergoing PD (HR = 1.47; 95% CI 1.01–2.11). Development of CAD was observed in 355 (25.28%) HD patients and 33 (15%) PD patients.

CAD, coronary artery disease; HD, hemodialysis; PD, peritoneal dialysis; ESKD, end stage kidney disease; ESRD, end-stage renal disease; CRP, C-reactive protein; HR, hazard ratio.

**Fig. 2.**
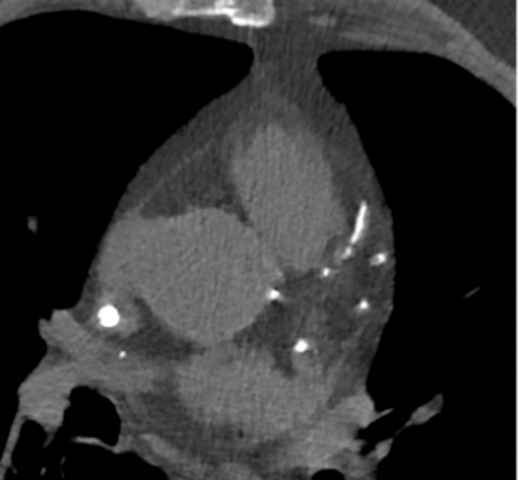
**Coronary artery calcifications observed in computed tomography (CT)**.

As previously mentioned, ESKD amplifies CAD risk as compared with the general population. The additional CV risk observed in CKD patients is mainly related to non-traditional risk factors. CAD is characterized by the presence of coronary artery calcification (CAC) due to calcium-phosphate crystal precipitation in the coronary vessel walls (Fig. 2). This process results from the imbalance between pro-calcification factors, such as fibroblast growth factor-23 (FGF-23) and sclerostin, and inhibitors such as Fetuin A, Matrix-Gla protein (whose activation depends on presence of vitamin K) and inorganic pyrophosphate (inhibited by bone alkaline phosphatase) [23, 24]. In turn, these alterations stimulate vascular smooth muscle cells (VSMCs) transdifferentiation into an osteoblast-like phenotype which are the leading process of vascular damage [25]. Vascular calcification can affect heart valves and vessels’ intima and media either in the heart (coronary artery) or in the large abdominal and peripheral vessels. Intimal calcifications are found in the context of atherosclerotic disease and preferably in medium and large caliber arteries with an abnormal flow (e.g., in vascular bifurcations) [26]. Although these calcifications are associated with traditional atherosclerotic risk factors, they are more severe in ESKD patients, demonstrating how kidney impairment can be considered an accelerator of atherosclerotic processes [27]. Medial calcifications, also known as Mönckeberg’s sclerosis, are typical of CKD patients and may develop in all vessels but more often in those where atherosclerotic lesions are unusual such as peripheral arteries (e.g., digital and radial). Medial calcifications are closely related to alterations in mineral metabolism and CKD, in fact, some studies have shown that in young patients undergoing dialysis, without traditional risk factors, only medial calcifications are detected [28]. The presence of CKD, at any stage, contributes significantly to the worsening of atherosclerotic processes. Autopsy studies on patients with CKD have shown that they have a higher degree of inflammation in the coronary plaques as compared to non-CKD patients [29]. Furthermore, an abnormal composition of extracellular matrix, which is observed in CKD patients where it associates with abnormal serum levels of matrix metalloproteinases (e.g., matrix metalloproteinases 2, matrix metalloproteinases 7 (MMP-2, MMP-7)), may contribute to accelerated plaque rupture and the onset of acute cardiac events [30].

Fluid overload is an emblematic complication in HD patients and often requires aggressive ultrafiltration; several studies have shown a relationship between fluid overload and increased CV risk [31]. Fluid overload is closely correlated with sodium intake which in turn promotes hypertension, left ventricular hypertrophy (LVH) and increased CV risk [32]. Control of sodium and fluids has shown beneficial effects on both hypertension and LVH. Using data from the Frequent Hemodialysis Network (FHN) Daily and Nocturnal trial, Raimann *et al*. [33] subdivided patients based on pre-dialysis sodium values (≥138 or <138 mEq/L) showing that the first group had a significant reduction in left ventricular mass [–28.0 (95% CI –40.5 to –15.4) g], compared to the group with sodium values >138 [–2.0 (95% CI –15.5 to 11.5) g)]. An interesting correlation was found in an observational study by Yu *et al*. [34], in which hippuric acid levels correlated significantly with LVH, in fact, patients who had LVH had higher hippuric acid levels (34.2 vs 18.1 µg/mL, *p* = 0.003). Moreover, LVH is a consequence of alterations in mineral metabolism and specifically of the increase in FGF-23 induced by an increase in parathormone (PTH) and hyperphosphatemia. In cardiomyocytes, PTH binds its parathyroid hormone 1 receptor (PTH1R) generating calcium influx within it which stimulates the phospholipase C pathway inducing myocardial hypertrophy [35]. A cross-sectional study on 3973 Japanese patients evaluated the association between mineral metabolism and CVD, demonstrating how cardiovascular mortality was significantly associated with PTH values (Relative risk: 1.08, *p* = 0.0001) [36]. An interesting analysis was performed by Xu *et al*. [37] on 72 HD patients undergoing cardiovascular magnetic resonance and comparisons with 30 healthy controls. Patients with PTH levels at target (150–300 pg/mL) or higher show increased myocardial damage, left ventricular mass and a decreased ejection fraction (*p* < 0.05) [37]. Furthermore, several studies, in vivo and in vitro, have documented how FGF-23, through a Klotho-independent mechanism mediated by FGFR4 that stimulates the PLCy phospholipase pathway, induces hypertrophic growth of cardiomyocytes and increases intracellular calcium levels and contractility of smooth muscle cells by promoting the expression of several genes linked to cardiac hypertrophy [38, 39]. Faul *et al*. [40] demonstrated that FGF-23 stimulates hypertrophy in isolated rat cardiomyocytes via Klotho-dependent and Klotho-independent pathways. Furthermore, the same authors, by measuring FGF-23 in a population of 3070 individuals participating in the Chronic Renal Insufficiency Cohort (CRIC) study, demonstrated how patients with high FGF-23 values had both a reduction in the ejection fraction and an increase in the mass of the left ventricle. Each one-unit increase in FGF-23 was associated with a 2.5-fold greater relative risk (RR) of eccentric hypertrophy and concentric hypertrophy (95% CI, 2.1–3.0; *p* < 0.001) [40]. The link between LVH and CAD is well demonstrated since patients with LVH are prone to have a larger infarct size and a higher risk of microvascular obstruction, a mechanism slightly different to atherosclerotic disease but one which significantly contributes to CAD [41].

Similarly, anemia is closely linked to LVH, cardiovascular and CAD risk. HD patients mostly present with hemoglobin values <10 g/dL, which leads to a reduction in afterload (lower viscosity and lower peripheral resistance) and an increase in preload (increased venous return). Altogether, these factors determine a greater activation of the sympathetic system with an increase in heart rate. These compensatory mechanisms eventually lead to left ventricle distension and LVH genesis [42]. The Association of Anemia, Iron parameters, and Mortality among the prevalent Hemodialysis patients (AIM-HD) study, retrospectively evaluated 42,230 patients from the Taiwan Renal Registry Data System (follow-up - 41 months), showing that hemoglobin values <10 g/dL were significantly associated with an increased risk for all-cause [hazard ratio (HR) 1.31 (95% CI 1.24–1.38), *p* = 0.001] and cardiovascular death [HR 1.23 (95% CI 1.15–1.32), *p* = 0.001]. In addition, ferritin levels between 300–800 ng/mL and transferrin saturation levels between 30–50% were associated with a low risk of mortality [43]. Conversely, a retrospective study on 252 patients with a 47 month follow-up showed not low hemoglobin values, but high variability in hemoglobin values had an increased risk of death from cardiovascular causes (*p* < 0.05) [44].

Another important non-traditional CV risk factor in ESKD patients is represented by uremic toxins, organic or inorganic substances that accumulate in the body fluids. Uremic toxins promote CV and CAD risk by endorsing oxidative stress and inflammation. There are more than 100 uremic toxins divided into protein-bound solutes, free water-soluble low-molecular-weight solutes (molecular weight <500 Da) and middle molecules (molecular weight ≥500 Da) [45]. Protein-bound uremic toxins derive mostly from the degradation of aromatic amino acids by the bacteria of the intestinal microbiome. These include indoxyl sulfate (IS), p-Cresyl Sulfate (pCS) and hippuric acid [46]. The underlying increasing CV risk mechanism is based on fibrosis and inflammation induction in the kidney as well as reactive oxygen species (ROS) synthesis stimulation which can lead to the progression of kidney disease and concomitant CV damage. Moreover, these solutes trigger endothelial dysfunction and inflammation and worsen vascular calcifications [47, 48]. The direct role of uremic toxins on heart tissue was also demonstrated. Indoxyl sulfate has been shown to have a pro-fibrotic and hypertrophic role in cardiomyocytes and cardiac fibroblasts [49]. Oxidative stress promotes CAD as well. Dimethylarginine (ADMA) is an endogenous nitric oxide synthase inhibitor which is increased in HD patients [50]. Increased plasma levels of ADMA are associated with vasoconstriction and hypertension and overall with an increased incidence of CAD. In particular, Zoccali and colleagues found that higher ADMA levels in blood were associated with about 15–20% of risk for CV events in HD patients [51]. The association between ADMA and CV risk may be at least in part explained by an ADMA-induced decrease in endothelial progenitor cell (EPC) circulating levels. The EPC, in turn, support endothelial cells and prevent atherosclerosis [52]. Another accelerating atherosclerosis trigger in patients with kidney impairment is the carbamylation of biochemical structures, in particular low-density lipoproteins (LDL). The LDL carbamylation promotes smooth muscle proliferation, a step of atherosclerotic plaque formation, and endothelial cell death [53].

Intradialytic events can be associated with CV events as well. Arterial hypotension is one of the most frequent complications during HD sessions. Sands *et al*. [54] in an observational study of 1137 patients recorded a 17.2% frequency of intradialytic hypotension (IDH) and observed a reduction in survival (*p* = 0.036) in those who had a higher frequency of intradialytic hypotension (>35% of treatments) compared to those who did not have episodes. Similarly, a 39.9% incidence of intradialytic hypotension was found in a single-center prospective study on 293 patients; after a 5-year follow-up the mortality rate was 5.2 per 100-person-year and CV events were 23%. After multivariate analysis, intradialytic hypotension and LVH were independent risk factors for long-term mortality [55]. Moreover, Mizuiri *et al*. [56] demonstrated that IDH is more frequently associated with CAC and patients with both IDH and CAC have a 3-year reduction in cumulative survival, a greater risk of CV events at 3 years, and a higher hazard ratio for CV mortality.

## 3. Prediction of CAD in Patients with ESKD

Prediction of fatal and non-fatal CV events, including CAD, in patients with kidney disease is a major goal of current research [57]. In fact, a true prediction of future events helps clinicians to refine treatment (being more aggressive in patients with higher predicted risk or less in those with lower risk), and schedule the appropriate number of screening visits in order to improve individual prognosis as much as possible. Unfortunately, there are two main limitations around this point. The first one is that the already available risk prediction models in patients with kidney disease are focused on ESKD (or progression in term of eGFR decline over time) as endpoint [58, 59]. A large part of individual risk prediction models have been calculated on the general population cohort but data on CKD patients followed by nephrologists are also published. Few data are, instead, forthcoming on the prediction of CV events rather than kidney events. The second issue is related to dialysis patients, where risk scores based on appropriate variables for patients at earlier CKD stages, do not discriminate accurately future events. Disparate attempts to improve CV risk prediction in patients with kidney disease have been made, with several markers of different pathophysiologic actions being tested. These encompass glomerular filtration markers (Cystatin C and beta2-microglobulin) and cardiac biomarkers (Troponin-T and probrain natriuretic peptide (BNP) [60, 61]. Across all the stages of CKD, eGFR based on Cystatin C has been shown to predict CV events including CAD with greater accuracy than creatinine-based eGFR. This is also true for patients immediately before dialysis starting.

Cardiac markers were found to be very useful in predicting CV and CAD events in CKD patients. Several studies have analyzed the role of the amino-terminal pro brain natriuretic peptide (NT-proBNP) demonstrating how it significantly correlates with the ultrasound detection of LVH and how it is important in risk stratification for the prediction of CV events. In a cohort of 8622 participants (of which 10.9% had eGFR <60 mL/min or albumin-creatinine ratio (ACR) >30 mg/g) during a follow-up of 11.9 years, the overall incidence rate of CV events was 38.0 per 1000 person-years in CKD patients (16.0 per 1000 person-years in non-CKD); this finding was strongly associated with the values of beta2-microglobulin, Troponin-T and pro B-type natriuretic peptide (pro-BNP) with HR 1.22 (95% CI 1.16–1.29), 1.61 (95% CI 1.43–1.81) and 1.50 (95% CI 1.34–1.68) respectively [62, 63, 64]. Unfortunately, pro-BNP and Troponin-T are affected by ESKD and impaired kidney clearance. In fact, patients on dialysis have slightly higher Troponin T values than the general population [65] and pro-BNP levels are 10 to 100 times higher than patients without CKD [66]. It has been hypothesized that this phenomenon does not derive from impaired clearance only but is associated with a subclinical cardiac pathology termed dialysis-induced myocardial stunning/myocardial strain [67]. Interestingly, it was demonstrated in 1152 dialysis patients, that the routine measurement of pro-BNP and Troponin-T is strongly associated with the risk of death and major cardiovascular events including CAD. Patients with elevated levels of cardiac markers were also older and had higher rates of CV disease, CAD and HF [68].

Other markers were evaluated in patients with ESKD. Among others, serum alkaline phosphatase (ALP) levels in a study evaluating 137 HD patients were significantly associated with CAC [69]. A meta-analysis of 10 studies with 2686 participants showed how the presence of vascular calcifications induces an increased risk of CV mortality (HR 2.81; 95% CI: 1.92–4.10) [70]. Moreover, a cross-sectional study enrolling 134 KF patients (94 undergoing HD and 30 PD), reported that vascular calcifications are not less present in PD patients than in HD ones; furthermore, the progression of calcifications had a delta of 1.72 per year in patients on HD (95% CI 0.81 to 2.64) and of 2.73 per year in patients on PD (95% CI 1.58 to 3.88) [71].

Other studies highlighted the role of FGF-23 in predicting the progression of CAC [72, 73], also independently from hyperphosphatemia, LVH and cardiac hypertrophy [74]. It has recently been shown that dialysis patients with higher interleukin-6 (IL-6) values experience greater progression than those with lower values [75]. This is likely due to the central role IL-6 plays in inflammation that promotes atherosclerosis and vascular calcification [76].

In parallel to the evaluation of serum biomarkers in ESKD patients, cardiac echocardiography demonstrated that LVH is a strong predictor of CV events [77, 78]. Paoletti *et al*. [79] evaluated 455 patients with CKD stage 2–5 demonstrating that the presence of concentric and eccentric LVH predicted the incidence of CV events and death. Furthermore, it has been demonstrated that LVH is associated with the reduction of coronary flow reserve (CFR); in fact, the increase in posterior wall thickness, evaluated with echocardiography, is a predictor of the reduction of CFR. This in turn predisposes individuals to an increased risk of myocardial infarction [80].

Similarly, carotid artery intima-media thickness (IMT) was evaluated in patients with ESKD demonstrating how these patients had a higher mean IMT and how this correlated with cholesterol and fibrinogen levels, both CV risk factors [81]. A recent meta-analysis has shown that IMT progression is associated with the risk of myocardial infarction (and other CV events such as stroke, and fatal CV disease) and all-cause mortality [82].

Risk stratification in ESKD patients is essential in order to prevent Major Adverse Cardiovascular Events (MACE) and to decide whether and when to use invasive methods. Furthermore, it is necessary for the evaluation of kidney transplant candidates; several studies have evaluated what might be the best method to stratify risk but currently, there are no conclusive data [83]. Some studies have used dobutamine stress echocardiography (DSE) which uses the properties of this substance to identify segmental or global ventricular dysfunction. Rakhit *et al*. [84], in a study of 224 CKD patients, including 169 on dialysis, classified as high or low risk via the Framingham risk score demonstrated that the high-risk group of patients had an impaired DSE and this predicted the outcome. Similarly, myocardial perfusion scintigraphy (MPS) was evaluated by Kim *et al*. [85] in a prospective study of 215 asymptomatic dialysis patients, divided into high and low risk according to clinical and echocardiographic parameters, demonstrated that high-risk patients with an abnormal MPS had a 3.3 greater relative risk of developing cardiac events compared to patients with normal MPS.

Hence, the utility of methods for evaluating vascular calcifications must be considered. The main CAC evaluating method is the Agatston score, also called the “calcium score”, which uses computed tomography [86]. This method, which has long been validated in the general population, has been evaluated in several studies in patients with ESKD. Raggi *et al*. [87] evaluated 205 HD patients and demonstrated that, in this cohort, the mean CAC score was significantly higher than in the general population and how it was directly related to the prevalence of myocardial infarction. Interestingly, the Agatston score was used to evaluate CAC in CRIC study population confirming the association between score and CV events as myocardial infarction and heart failure [88]. It should be emphasized that the role of this score in the CKD population has long been debated, as some small studies had not confirmed these associations, while it is currently believed to have a greater prognostic value. In fact, although it is less specific for the presence of atherosclerosis, it could be a marker of vascular calcification related to typical alterations of CKD, even if it does not distinguish between medial and intimal calcifications [88]. Other methods of evaluating vascular calcification are the Kauppila score which measures abdominal aortic vascular calcifications [89] and the Adragao score which evaluates calcifications of the iliac arteries and lower abdominal aorta [90].

## 4. Therapeutic Strategies for CAD in ESKD Patients

Treatment of CAD is a cornerstone to reducing CV risk in ESKD patients. These patients should be considered at high CV risk; recently, dialysis has been classified as equivalent CV risk like diabetes or previous CV disease history. Therapeutic strategies include lifestyle changes, pharmacological therapies, surgical interventions, and high-quality kidney replacement treatment (Table 2, Ref. [91, 92, 93, 94, 95, 96, 97, 98, 99, 100, 101, 102]). Nowadays, optimal medical management of CAD cannot be dissociated from lifestyle counseling and recommendations focused on daily habits changes. Among these, smoking is notoriously recognized as one of the main CV risk factors and, regardless of the number of cigarettes per day, it is strongly associated with a higher incidence of acute coronary syndrome and consequently with a higher risk of sudden cardiac death [103]. Therefore, smoking cessation reduces this risk and it is associated with an improvement in endothelial dysfunction [104].

**Table 2.** **Randomized clinical trials regarding therapeutic strategies for CAD in patients with advanced CKD and ESKD**.

Pharmacological interventions					
Study	Population	Sample size	Intervention	Outcome	Results
4D study [91]	Type 2 diabetes mellitus and ESRD on dialysis	1255; Atorvastatin 20 mg (n = 619) or placebo (n = 636)	Atorvastatin 20 mg versus placebo	Composite variable of death from cardiac causes, fatal or nonfatal stroke, nonfatal myocardial infarction	Atorvastatin had no statistically significant effect on CV death, nonfatal myocardial infarction and stroke
AURORA study [92]	ESKD on hemodialysis	2776; Rosuvastatin 10 mg or placebo	Rosuvastatin 10 mg versus placebo	Composite variable of death from CV causes, nonfatal myocardial infarction, or nonfatal stroke	Rosuvastatin did not show significant effect on the composite primary end point of death from CV causes, nonfatal myocardial infarction, or nonfatal stroke
Treat-To-Goal study [93]	ESKD on hemodialysis with hyperphosphatemia	200; Sevelamer 800 mg (n = 99) or calcium-based phosphate binders (n = 101)	Sevelamer 800 mg versus calcium acetate 667 mg or calcium carbonate 500 mg	Target concentrations of serum phosphorus, calcium, PTH, and calcification of the coronary arteries and thoracic aorta	Treatment with sevelamer did cause fewer episodes of hypercalcemia, improved control of PTH and attenuation of the progression of coronary artery and aortic calcification
Fujii, 2017 [94]	ESKD on hemodialysis	105; Lanthanum carbonate (n = 50) or calcium carbonate (n = 55)	Lanthanum carbonate versus calcium carbonate	Improvement of CAC and cardiac abnormalities	Lanthanum improved cardiac dimension and systolic function, and improved CAC in patients with moderate CAC
IMPROVE-CKD study [95]	CKD in stage III and IV and normophosphatemia	278; lanthanum 500 mg (n = 138) or placebo (n = 140)	Lanthanum 500 mg versus placebo	Carotid-femoral pulse wave velocity, abdominal aortic calcification and serum and urine markers of mineral metabolism	Lanthanum did not affect arterial stiffness or aortic calcification compared with placebo
EPISODE study [96]	ESKD on dialysis	115; Lanthanum carbonate (n = 62) or sucroferric oxyhydroxide (n = 53)	Lanthanum carbonate vs sucroferric oxyhydroxide	Percentage change in coronary artery calcification (CAC) scores during 12-month treatment	Phosphate binders did not differ in their effect on CAC progression
SPACE study [97]	ESKD on hemodialysis	196; Vitamin E 800 IU/day or placebo	Vitamin E 800 IU/day versus placebo	Composite variable of myocardial infarction, ischaemic stroke, peripheral vascular disease and unstable angina	Supplementation with vitamin E reduces composite CV disease endpoints and myocardial infarction
Tepel, 2003 [98]	ESKD on hemodialysis	134; Acetylcysteine 600 mg BID (n = 64) or placebo (n = 70)	Acetylcysteine 600 mg BID versus placebo	Composite variable of myocardial infarction, CV disease death, need for coronary angioplasty or coronary bypass surgery, ischaemic stroke, peripheral vascular disease with amputation or need for angioplasty	Treatment with acetylcysteine reduces composite CV endpoints
MAGiCAL-CKD study [99]	CKD in stage IV and IIIb	148; Magnesium hydroxide 15 mmol twice daily (n = 75) or placebo (n = 73)	Magnesium hydroxide versus placebo	Difference in coronary artery calcification score after 12 months	Magnesium did not slow the progression of vascular calcification in CKD
**Interventional strategies**					
CREDO-Kyoto study [100]	ESKD with multivessel and/or left main coronary artery disease	388; PCI (n = 258) or isolated CABG (n = 130)	PCI versus CABG	Difference in 30-day mortality, 5-year all-cause mortality, risk of cardiac death, sudden death, myocardial infarction and any revascularization	CABG reduced the risk of cardiac death, sudden death, myocardial infarction and any revascularization
ISCHEMIA-CKD study [101]	CKD stage IV and V, stable coronary artery disease, and moderate or severe ischemia	777; Initial invasive strategy and medical therapy (n = 388) versus initial medical therapy alone and angiography if medical therapy failed (n = 389)	Cardiac catheterization and revascularization with optimal medical therapy versus conservative strategy of optimal medical therapy	Composite of death or nonfatal myocardial infarction	Patients undergoing invasive procedures did not show better outcomes than patients receiving initial conservative medical therapy
**Renal replacement therapies**					
CONVINCE trial [102]	ESKD on hemodialysis	1360; High-dose hemodiafiltration (n = 683) versus high-flux hemodialysis (n = 677)	High-dose hemodiafiltration vs high-flux hemodialysis	Difference in death from any cause	High dose-hemodiafiltration was associated with a lower mortality rate from any cause (HR 0.77, 95% CI 0.65–0.93)

CABG, coronary artery bypass grafting; CAC, coronary artery calcification; CKD, chronic kidney disease; ESRD, end-stage renal disease; PCI, percutaneous coronary intervention; CAD, coronary artery disease; 4D, Die Deutsche Diabetes Dialyze Studie; AURORA, A Study to Evaluate the Use of Rosuvastatin in Subjects On Regular Haemodialysis: an Assessment of Survival and Cardiovascular Events; IMPROVE-CKD, Effect of Phosphate Reduction on Vascular End Points in CKD; EPISODE, Evaluate the New Phosphate Iron-Based Binder Sucroferric Oxyhydroxide in Dialysis Patients with the Goal of Advancing the Practice of EBM; SPACE, Secondary prevention with antioxidants of cardiovascular disease in endstage renal disease; MAGiCAL-CKD, Magnesium Supplementation on Vascular Calcification in CKD; CREDO-Kyoto, Coronary REvascularization Demonstrating Outcome Study in Kyoto; ISCHEMIA-CKD, International Study of Comparative Health Effectiveness With Medical and Invasive Approaches-Chronic Kidney Disease Trial; CONVINCE, Comparison of high-dose HDF with high-flux HD; ESKD, end stage kidney disease; CV, cardiovascular; PTH, parathyroid hormone; BID, bis in die; HR, hazard ratio.

Also, dietary recommendations are an essential part of the counseling. On one hand, a hypocaloric diet allows weight control in cases of obesity and large waist circumferences, which are predictors of CV disease and mortality in patients with CKD [105]; in fact, KDIGO (Kidney Disease: Improving Global Outcomes) guidelines recommend a body mass index value between 20 and 25 ${\mathrm{kg/m}}^{2}$ as a target reference [106]. On the other hand, limiting dietary sodium intake to 2 g (90 mmol) per day and phosphorus intake to 800 mg per day represent useful tools to respectively improve blood pressure control secondary to fluid overload [107, 108] and hyperphosphatemia in dialysis patients [109].

Moreover, regular physical aerobic activity of at least 30 minutes per day is highly recommended in CKD patients [110]: a sedentary lifestyle is widely recognized as a risk factor for higher morbidity and mortality and an international survey showed that about 45% of patients with ESKD do not perform any activity at all [111]. In fact, physical exercise not only improves physical and mental performance but is also associated with better blood pressure control, and glycemic balance in diabetic patients, and it is inversely correlated with all-cause death and CV mortality [112]. A recent multicenter randomized clinical trial, the EXerCise Introduction to Enhance performance in dialysis (EXCITE) trial, which enrolled HD patients, demonstrated that a home-based low-intensity exercise program such as walking exercise improves functional status and quality of life [113]: physical performance at 6 months assessed that the exercise group improved the distance covered during the 6-minute walking test (6-MWT) (baseline, 328 ± 96 m; 6 months, 367 ± 113 m) and reduced the time to perform the 5 times sit-to-stand test (5xSTS) (baseline, 20.5 ± 6.0 seconds; 6 months, 18.2 ± 5.7 seconds), while the control arm which performed normal physical activity did not show any improvement (*p* < 0.001 between groups). In addition, a sub-analysis of the EXCITE trial demonstrated that a 20 m increase in the 6-MWT resulted in a 6% reduction (*p* = 0.001) of the risk of the composite endpoint (i.e., mortality, fatal and non-fatal cardiovascular events and hospitalizations) [114].

Although several lifestyle interventions contribute to reduced CV risk, the clinical complexity of dialysis patients also requires pharmacological interventions and sometimes more invasive strategies. Medical management of stable CAD aims to modify the natural history of the disease and include anti-platelet agents, beta-blockers, calcium channel blockers, statins, phosphate-binders, secondary hyperparathyroidism therapies, and angiotensin-converting enzyme (ACE)-inhibitors/angiotensin receptor blockers (ARBs). However, despite patients with ESKD showing a significantly higher risk of CV events and mortality compared to the general population, they are notoriously under-represented in most randomized clinical trials concerning CAD [115], creating significant controversies around the benefit of specific treatments in this niche category of patients and justifying the diffusion of “therapeutic nihilism” that many clinicians caring for dialysis patients may experience [116]. Interestingly, Berger *et al*. [117] examined the efficacy and the rate of aspirin usage, beta-blockers and ACE inhibitors in patients with ESKD facing an acute myocardial infarction: analyzing the data from the ESKD database and the Cooperative Cardiovascular Project database, the Authors concluded that ESKD patients are less likely to be treated with these medications with a consequent increased mortality, compared to non-ESKD patients; this is quite contrasting since, if treated with aspirin after acute myocardial infarction (AMI), dialysis patients showed a reduction of mortality of 43% after 30 days.

Among the modifiers of natural disease history of CAD, anti-hypertensive drugs play a central role: even though unique blood pressure target recommendations on dialysis patients do not exist, the reference thresholds of 130/80 mmHg from the 2017 American College of Cardiology/American Heart Association Guidelines and systolic blood pressure (SBP) target of <130 mmHg for patients younger than 65-year-old and SBP target range of 130–140 mmHg for all the others from the 2018 European Society of Hypertension/European Society of Cardiology Guidelines are widely accepted [118, 119]. On the other hand, avoiding hypotension, especially intradialytic, is paramount for the associated higher risk of vascular access thrombosis, dialysis inadequacy and higher mortality rate [120]. A retrospective analysis conducted by McCullough and colleagues showed that the use of ACE inhibitors after cardiac events conferred a significant all-cause mortality reduction (HR 0.63, 95% CI 0.47–0.83, *p* = 0.001) over long-term survival of patients with ESKD [121]. Similarly, Winkelmayer *et al*. [122] assessed that ACE inhibitors and ARBs were associated with the reduction of mortality risk at 1 year after AMI (HR 0.70, 95% CI 0.50–0.98). These results were in contrast to those from the secondary analysis of the HEMO study conducted by Chang *et al*. [123], which did not reveal any significant association between usage of ACE inhibitors and mortality (HR 0.97%, 95% CI 0.82–1.14).

Concerning lipid-lowering agents, the 4D Study trial (Deutsche Diabetes Dialyze Studie) demonstrated the safety and efficacy of atorvastatin to reduce LDL cholesterol in ESKD patients, however, investigators revealed a non-significant reduction of the primary end-point (composite of cardiac death, nonfatal AMI, stroke) by only 8% (*p* = 0.37) [91]. Similar findings were reported in the Assessment of Survival and Cardiovascular Events (AURORA) study [92]. These well-conducted trials examined the efficacy of statins in reducing the death risk from CV causes, nonfatal myocardial infarction (MI), and stroke. Although no benefit overall, post hoc analyses later showed a protective effect of statins in the category of HD patients with previous myocardial infarction [124]. These support strict attention to lipid panels in these patients, especially those with high CV risk.

In the evaluation of the role of phosphate binders over coronary artery disease, the randomized Treat-To-Goal trial demonstrated an attenuation of the progression of CAC with sevelamer relative to calcium salts such as calcium carbonate and calcium acetate [93]. Also, lanthanum carbonate was shown to ameliorate cardiac abnormalities (such as cardiac dimension and systolic function) and to improve the CAC score in patients with moderate calcifications at 18 months of therapy, compared to calcium carbonate [94], however without improving arterial stiffness or aortic calcification in patients with normal phosphatemia [95]. Moreover, no significant differences were found between lanthanum and sucroferric oxyhydroxide in their effect on CAC progression [96].

Regarding antioxidants agents, that may reduce the oxidative stress above mentioned, vitamin E administration has been shown to significantly reduce, as compared to placebo, the incidence of CV events including CAD in a randomized study enrolling 196 HD patients [97]. A similar result in the reduction in CV mortality was also demonstrated by the use of high dose acetylcysteine (1200 milligrams per day) [98]. Contrastingly, magnesium supplementation did not slow the progression of CAC [99].

The main limitation of all the randomized studies on CAD and CV risk in HD patients is the lack of large samples and a convincing reproducibility of the results to individual patients.

Invasive therapeutic strategies of revascularization include coronary artery bypass grafting (CABG) and percutaneous coronary intervention (PCI). Recent studies have shown that revascularization interventions in dialysis patients improve the survival of these patients [125]. Among these procedures, CABG seems to be associated with more favorable outcomes than PCI. Interestingly, a japanese analysis performed on 388 KF patients undergoing dialysis from the Coronary REvascularization Demonstrating Outcome Study in Kyoto (CREDO-Kyoto) PCI/CABG Registry Cohort-2 showed a superiority in terms of reduction of 5-year mortality of CABG compared to PCI (49.9% vs 52.3%, respectively). Furthermore, patients treated with CABG showed a significant reduction in the risk of cardiac death, myocardial infarction and necessity for any other coronary revascularization than after PCI [100]. However, recent randomized clinical trials demonstrated an overlap in terms of outcomes between intensive medical therapy (optimization of lifestyle, smoking cessation, antiplatelet therapy, beta-blockers, ACE-inhibitors (ACEi)/ARB, statins, etc.) and routine revascularization in patients with stable CAD, as evidenced by the COURAGE (Clinical outcomes utilizing revascularization and aggressive drug evaluation) [126], BARI-2D (bypass angioplasty revascularization investigation in type 2 diabetes) trials [127] and ISCHEMIA (International Study of Comparative Health Effectiveness With Medical and Invasive Approaches) [128] studies. However, these results were limited since patients with advanced CKD were underrepresented in these trials. More specifically, the ISCHEMIA-CKD (International Study of Comparative Health Effectiveness With Medical and Invasive Approaches-Chronic Kidney Disease Trial) assessed the initial invasive approach added to medical therapy did not show any incremental benefit than the conservative strategy in patients with advanced CKD and stable CAD [101].

Conversely, several studies concerning kidney replacement therapy have demonstrated a significant survival benefit of high-dose hemodiafiltration. The results of the multicenter international CONVINCE (Comparison of high-dose HDF with high-flux HD) trial, which randomized 1360 KF patients to hemodiafiltration and conventional high-flux HD, showed that the usage of high-dose hemodiafiltration (guaranteeing at least 23 liters of convection volume per session) was associated with a lower mortality rate from any cause (HR 0.77, 95% CI 0.65–0.93) compared to conventional standard high-flux HD [102]. Similarly, a pooled retrospective analysis conducted by Peters and colleagues revealed that online hemodiafiltration reduced the risk of CV death by 31% than HD [129].

However, two aspects must be taken into account: first, the generalizability of the results of CONVINCE trial to the general dialytic population may be limited due to the relatively young age of enrolled patients (mean age of 62.5 years) and the high percentage of arteriovenous fistula (>80%); second, hemodiafiltration is a convective and diffusive technique which requires specific and high-costly filters and dialysis machine, therefore its use is limited to high-income industrialized countries, like European countries and USA.

## 5. Dialysis as Model of Care and Research

On the basis of epidemiological and prognostic data, we contend that patients with KF represent a population that needs more attention on behalf of clinicians and the scientific community. The status of very-high CV risk and the extremely poor outcome of patients with KF led to the well known therapeutic nihilism [11]. This phenomenon was mainly driven by the lack of robust evidence about efficacy of treatments, dialysis modality, diet, and lifestyle changes on improving future outcomes, so that residual risk remains strikingly high. Nephrologists adapted treatments in KF patients based on guidelines from the general population or empirical strategies, which certainly do not lead to brilliant results, especially if compared to settings with active research and available trials [130, 131]. In the past few years, the tendency seemed to change. One important example is given by the completion of randomized studies assessing the efficacy of disparate interventions in dialysis patients: the role of cognitive therapies on depressive symptoms in HD patients, given the fact that depression belongs to the major outcomes of dialysis patients being directly related to the future risk for death [132]; the improvement in nutritional status in HD patients after administration of low protein calorie supplements [133]; the effect of exercise intervention on the 60-second sit-to stand test in HD patients, this latter on a topic with none evidence until 2020 [134, 135]. Importantly, a recent randomized study enrolling more than 1300 patients undergoing HD, proved the significant reduction in mortality risk with hemodiafiltration compared to standard HD, confirming previous similar evidence specifically on CV mortality [129]. However, a big effort is still needed to clarify what is the best screening for CV disease and CAD in HD patients and the following treatment options [136]. This is also true for treatment with a historical and consolidated protective efficacy in the general population or earlier stages of CKD such as statins and blood pressure lowering therapies.

If from one side novel research also means novel efforts in terms of personnel and funding, the advantage to carry out clinical and preclinical studies in dialysis patients is their feasibility in terms of blood samples as well as patients availability. The fact that patients reach the clinic for therapy every three days, makes the study variables collection easier, always keeping in mind the ethical and scientific importance of the research question. Another important point is that the study of patients during dialysis allows for the evaluation of changes in serum levels of a given biomarker across dialysis sessions (pre vs post sampling) [9, 137]. Following this interesting approach, a recent study by Bolignano and colleagues showed that marinobufagenin (MBG), a steroidal marker of CV disease, was stable across HD sessions and significantly modified by episodes of intradialytic hypotension, and can be thus be proposed in the future as a biomarker useful to reduce hypotensive episodes which are in turn associated with CV mortality [138]. Similar analyses should be run testing the variability of troponins and pro-BNP, among other biomarkers, where evidence is lacking thus far. These shortcomings in KF patients also claim the need for a reliable individual CV risk prediction model, built specifically in these patients. The individual risk prediction model derives from a combination of biomarkers that, taken together, predict accurately an outcome. You and colleagues built a prediction model of CV disease in a cohort of about 400 dialysis patients in China and found that age, hypertension, diabetes and abnormal white blood cell counts were significant predictors of the outcome [139]. Moreover, Kanda *et al*. [140] created an individual risk prediction model of mortality specifically in HD patients considering age, body mass index, serum creatinine, albumin, total cholesterol and phosphorus levels, history of cardiovascular diseases, and arteriovenous fistula use, and reported a very good prediction ability with a c-index of 0.74. The same authors used artificial intelligence to create different clusters of mortality based on kidney diagnosis, age and nutritional factors expanding the potential factors to be included in patient evaluations [140]. Future risk prediction models that are also more reproducible across races and Countries are eagerly expected as well as prediction models that optimally stratify the risk for CAD.

A summary of the article’s main evidence is reported in Table 3.

**Table 3.** **Summary of the key-messages of the present Review**.

	Key-Messages
ESKD and CAD	∙ CKD is associated with an increased risk of CV disease and CAD. As eGFR goes below 60 mL/min the risk of CAD increases linearly and reaches the highest values in ESKD patients.
	∙ There are the traditional risk factors to which they add non-traditional risk factors related to CKD (mineral bone disorder, anemia, oxidative stress, inflammation, left ventricular hypertrophy, vascular calcification) and in ESKD those related to dialysis session (type, frequency, intradialytic hypotension, myocardial stunning and continuous changes in extracellular and intravascular body volume).
Prediction of CAD in ESKD	∙ Laboratory tests: Strong associations have been found between CV events and Troponin-T and pro-BNP, between ALP and CAC, and between FGF-23 and LVH.
	∙ Ultrasound: the presence of LVH and the positivity of the DSE is closely related to the risk of CV events. There is a correlation between carotid IMT and CV risk factors.
	∙ Imaging: detection of CAC through the Agatson score is associated with CAD and CV events.
Treatment of CAD in ESKD	∙ Lifestyle changes: smoking cessation, diet, regular physical activity have shown to protect from CAD risk.
	∙ Drugs: anti-platelet agents, beta-blockers, calcium channel blockers, statins, and ACE-i/ARB, antioxidants decrease CAD risk in HD patients even if further and more robust evidence are needed.
	∙ Invasive therapeutic strategies: CABG seems to be associated with more favorable outcomes than PCI.
Research perspectives	∙ Implementation of prognostic biomarkers of CAD and individual risk prediction models of CAD events in HD patients is eagerly expected, since the available score used in general population are not applicable.
	∙ Randomized studies testing the efficacy of disparate drugs in reducing CAD risk on a large number of patients are needed.
	∙ HD is a peculiar setting that offers the opportunity to implement research and a strict monitoring of patients conditions.

ACE-i, angiotensin converting enzyme inhibitors; ARB angiotensin receptor blockers; BNP, B-type natriuretic peptide; CABG, coronary artery bypass grafting; CAC, coronary artery calcification; CAD, coronary artery disease; CKD, chronic kidney disease; CV, cardiovascular; eGFR, estimated glomerular filtration rate; ESKD, end-stage kidney disease; FGF-23, fibroblast growth factor-23; HD, hemodialysis; LVH, left ventricular hypertrophy; PCI, percutaneous coronary intervention; ALP, alkaline phosphatase; DSE, dobutamine stress echocardiography; IMT, intima-media thickness.

## 6. Conclusions

Recent improvements in CV risk management in KF patients encourage continuing to work with strength and motivation. HD patients represent a high-risk population but, from an epidemiologic perspective, they are a dynamic population too. In fact, the average duration of HD before kidney transplant is changing and the health presentation to the transplant itself should be brought to the attention of clinicians. Future efforts should involve the prognostic research namely finding biomarkers that predict CV event risk in the HD population, and intervention studies, i.e., those testing the efficacy of old and novel treatments proving the efficacy in reducing CAD risk specifically in HD patients. These strategies, combined with the shortening of HD duration, may reduce CV risk and mortality in HD patients, helping clinicians plan of kidney transplants as well as the routine care.
